# A Hybrid Bottom-Up and Data-Driven Machine Learning Approach for Accurate Coarse-Graining of Large Molecular Complexes

**DOI:** 10.1021/acs.jctc.5c00063

**Published:** 2025-04-17

**Authors:** Korbinian Liebl, Gregory A. Voth

**Affiliations:** Department of Chemistry, Chicago Center for Theoretical Chemistry, Institute for Biophysical Dynamics, and James Franck Institute, 2462 The University of Chicago, Chicago, Illinois 60637, United States

## Abstract

Bottom-up coarse-graining refers to the development of low-resolution simulation models that are thermodynamically consistent with certain distributions from fully atomistic simulations. Force-matching and relative entropy minimization represent two major, frequently applied methods that allow to develop such bottom-up models. Nevertheless, atomistic simulations can often provide only limited sampling of the phase space. For bottom-up coarse-graining, these limitations may result in overfitting of the atomistic reference data, especially for large molecular complexes, where the learning may be agnostic of the actual affinities between binding partners. As a solution to this problem, we devise a data-driven machine learning hybrid coarse-graining concept that represents a regularized version of the relative entropy minimization approach. We demonstrate that this new approach allows one to develop coarse-grained models for molecular complexes that reproduce the targeted binding affinity but also describe the underlying complex structure accurately. The trained models therefore show diverse behavior as they can undergo frequent unbinding and binding events and are also transferable for simulating entire protein lattices, e.g., for a virus capsid.

## Graphical abstract

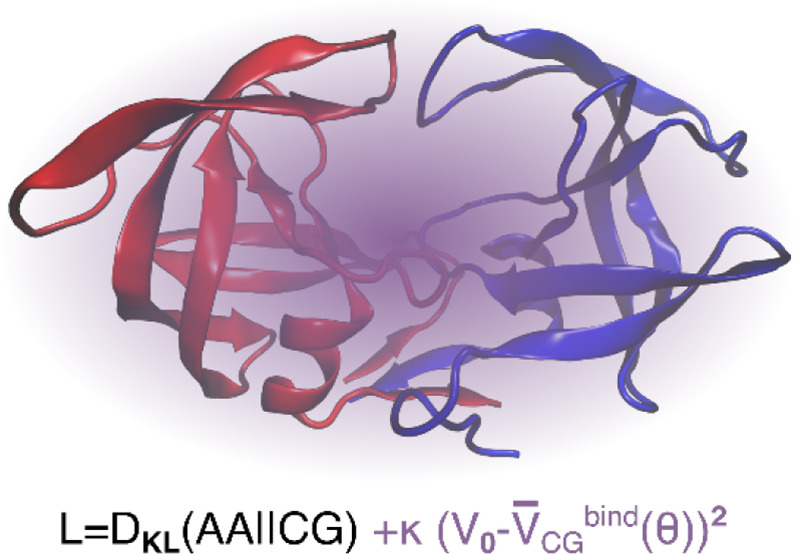

## Introduction

Molecular Dynamics (MD) simulations have gained substantial popularity, as they facilitate atomistic insight into the dynamics of biomolecules.^1–4^ MD simulations are at present routinely performed on the microsecond time scale, and atomistic MD simulations of virus-systems consisting of up to 100 million atoms have been carried out.^5–7^ Moreover, advanced free energy simulation techniques also allow one to capture free energy barriers associated with significantly slower events and to quantify the underlying energy-landscape.^8–12^ For larger systems with an intricate dynamics, however, such simulations are difficult to design, computationally too expensive, and the atomistic resolution may also be dispensable.

For these reasons, coarse-grained (CG) simulations have emerged to address large-scale simulation problems.^13–15^ CG models follow from defining mapping rules between atomistic and CG structures and from a parametrization of the effective potential energy function in the CG variables. Ideally, a CG-model is thermodynamically consistent with the CG-projected atomistic ensemble, otherwise it may suffer from, e.g., an erroneous entropy-enthalpy decomposition and to then display behavior inconsistent with statistical mechanics as applied to the underlying atomistic model.^16–18^ CG-models trained toward thermodynamic consistency with atomistic data are often referred to as “bottom-up” models.^13^

Multiscale Coarse-graining (MS-CG) (aka “Force-matching” or FM)^19–21^ and Relative Entropy Minimization (REM)^22–24^ are two major methods to parametrize the CG-interactions in a bottom-up approach. In FM, the CG-parameters are derived from variational minimization of the mean-squared deviation between a candidate CG-force field and atomistic forces mapped onto the CG-beads.^20,21^ As has been recently pointed out, the mapping of the forces is not strictly defined, but it can be optimized to reduce statistical uncertainties.^25^ In addition, FM-based learning of the CG force field can be improved through a neural-network parametrization or sampling from normalizing flows.^26–28^ In REM, the CG parameters are iteratively updated to minimize the Kullback–Leibler (KL) divergence between the atomistic and the coarse-grained statistics.^22^ Over the past few years, REM-based models have achieved noticeable success, for instance, in the self-assembly of mixed lipid bilayers,^29^ as well as the HIV capsid lattice and its translocation into the nuclear pore.^30^

However, the accuracy of bottom-up coarse-grained models can be limited by the sampling in the underlying atomistic simulation as one might expect since these are at their core data-driven machine learning models (without or with neural networks to represent them). Such a limitation may be particularly problematic for biomolecular complexes, where the limited time scale of atomistic simulations can rule out convergence toward a bound-unbound state equilibrium. Thus, we assert that standard bottom-up coarse-graining for biomolecular complexes likely results in significant overfitting of the training data (atomistic MD simulation data), and consequently in exaggerated binding affinities.

To address this problem, we present a solution to regularize the REM-based KL-minimization by biasing the average interaction energy between binding partners toward a “known” value (which may even be empirical or hypothetical). We demonstrate this new hybrid coarse-graining approach for three systems: The HIV-protease dimer, the CA/SP1 immature Gag lattice, and a four base-pair DNA duplex. We obtain an excellent structural description (similar to standard REM) but also achieve the targeted binding affinity for all three complexes. Furthermore, we show that such models can then be used to conduct robust CG simulations for HIV protein lattices. The usefulness of our method also becomes particularly obvious for the weakly binding DNA oligomers. At elevated temperature, the CG-model parametrized with the new method shows frequent binding and unbinding of the two molecules. Thus, the proposed regularized relative entropy minimization (reg-REM) method is seen to provide a reliable protocol to develop CG models that accurately capture the molecular structure but also offer a realistic description of the protein complex binding-affinity. In this way, the reg-REM method will facilitate a multitude of future CG studies of large-scale multiprotein structures and their formation processes.

## Theory and Methodology

### The Relative Entropy Minimization Method

The relative entropy between an atomistic and coarse-grained model can be expressed as^22^

$$S_{rel}=\int dr^{n}p_{AA}\left(r^{n}\right)\cdot \ln\left(\frac{p_{AA\left(r^{n}\right)}}{p_{CG}\left(M\left(r^{n}\right)\right)}\right)+\int dr^{n}p_{AA}\left(r^{n}\right)\cdot \ln\left(\Omega_{map}\left(M\left(r^{n}\right)\right)\right)$$ 1

where *M*(**r** ^*n*^) maps from atomistic to coarse-grained coordinates and *Ω*_*map*_ accounts for the degeneracy in this mapping. Here, *p*_*AA*_ and *p*_*CG*_ are the probability distributions of the atomistic and coarse-grained models, respectively. Note that the last term in eq 1 is independent of *p*_*CG*_ and hence of the coarse-grained effective potential energy *U*_*CG*_(***R*** ^N^,**θ**). Thus, the effective loss-function (*L*) for the optimization of the CG model parameters **θ** is the first term in eq 1 which is the Kullback–Leibler divergence^31^ between the atomistic and coarse-grained model (*D*_*KL*_(*AA*||*CG*)). In the canonical ensemble, we therefore obtain

2 $$\nabla_{\theta }L=\beta \langle \nabla_{\theta }{U_{CG}\rangle }_{AA}-\beta \langle \nabla_{\theta }{U_{CG}\rangle }_{CG}$$

The CG model parameters can then be iteratively refined via

3 $$\theta_{i+1}=\theta_{i}-\gamma \nabla_{\theta }L$$

for instance, with γ denoting the learning rate for this machine learning problem. (We note that in the two equations above and in subsequent expressions the set of CG model parameters **θ** is denoted in nonbold face form as θ.)

### Definition of the Problem

Accurate training of the CG-parameters critically depends on a sufficient sampling-set of the atomistic simulation (eq 2). Typical atomistic simulations, however, are on the low-microsecond time-scale. The consequences of this limitation are system dependent, but may be particularly serious for molecular complexes, where the atomistic simulations generally fail to capture multiple unbinding/binding events and therefore cannot encode a realistic affinity of the binding partners. In practice, atomistic reference simulations are mostly conducted starting from the bound structure which never dissociates throughout the simulations. Training a CG-model based on such a data set therefore tends to greatly overestimate the affinity of the binding partners. In conclusion, standard REM-procedures (eq 2) can overfit the training data resulting in an unrealistic description of the stability of molecular complexes.

### Solution to the Problem

To address the overfitting problem, we suggest to “regularize” the KL-divergence by biasing the average CG interaction energy between binding partners $\left({\mathrm{V̅}}_{CG}^{bind}\right)$ toward an input or empirical value *V*_0_. The loss-function in this approach is then given by

4 $$L=D_{KL}\left(AA\left|\right|CG\right)+\kappa \cdot {\left(V_{0}-{\mathrm{V̅}}_{CG}^{bind}\left(\theta \right)\right)}^{2}$$

with κ being a constant regularization strength. The ${\mathrm{V̅}}_{CG}^{bind}$ term is defined within the CG-space and unaffected by the atomistic data. It represents the average of the CG potential energy due to the interactions between the binding partners and can be expressed as an ensemble average. The gradient on the regularization term is calculated as follows:

5 $$\nabla_{\theta }\kappa \cdot {\left(V_{0}-{\mathrm{V̅}}_{\mathrm{CG}}^{\mathrm{bind}}\left(\theta \right)\right)}^{2}=-2\kappa \cdot \left(V_{0}-{\mathrm{V̅}}_{\mathrm{CG}}^{\mathrm{bind}}\left(\theta \right)\right)\nabla_{\theta }\frac{\int dR^{N}V_{\mathrm{CG}}^{\mathrm{bind}}\left(\theta \right)\cdot \exp\left(-\beta U_{\mathrm{CG}}\left(\theta \right)\right)}{\int dR^{N}\exp\left(-\beta U_{\mathrm{CG}}\left(\theta \right)\right)}=-2\kappa \cdot \left(V_{0}-{\mathrm{V̅}}_{\mathrm{CG}}^{\mathrm{bind}}\left(\theta \right)\right)\cdot \left[\left\{\int dR^{N}\left(\nabla_{\theta }V_{\mathrm{CG}}^{\mathrm{bind}}\left(\theta \right)\right)\cdot \exp\left(-\beta U_{\mathrm{CG}}\left(\theta \right)\right)-\int dR^{N}V_{\mathrm{CG}}^{\mathrm{bind}}\left(\theta \right)\cdot \beta \exp\left(-\beta U_{\mathrm{CG}}\left(\theta \right)\right)\nabla_{\theta }U_{\mathrm{CG}}\left(\theta \right)\right\}/\left\{\int dR^{N}\exp\left(-\beta U_{\mathrm{CG}}\left(\theta \right)\right)\right\}+\left\{\left[\int dR^{N}\beta \exp\left(-\beta U_{\mathrm{CG}}\left(\theta \right)\right)\nabla_{\theta }U_{\mathrm{CG}}\left(\theta \right)\right]\cdot \left[\int dR^{N}V_{\mathrm{CG}}^{\mathrm{bind}}\left(\theta \right)\cdot \exp\left(-\beta U_{\mathrm{CG}}\left(\theta \right)\right)\right]\right\}/\left\{{\left[\int dR^{N}\exp\left(-\beta U_{\mathrm{CG}}\left(\theta \right)\right)\right]}^{2}\right\}\right]=-2\kappa \cdot \left(V_{0}-{\mathrm{V̅}}_{\mathrm{CG}}^{\mathrm{bind}}\left(\theta \right)\right)\cdot [{\left\langle \nabla_{\theta }V_{\mathrm{CG}}^{\mathrm{bind}}\left(\theta \right)\right\rangle }_{\mathrm{CG}}-\beta {\left\langle V_{\mathrm{CG}}^{\mathrm{bind}}\left(\theta \right)\cdot \nabla_{\theta }U_{\mathrm{CG}}\left(\theta \right)\right\rangle }_{\mathrm{CG}}+\beta {\left\langle \nabla_{\theta }U_{\mathrm{CG}}\left(\theta \right)\right\rangle }_{\mathrm{CG}}{\left\langle V_{\mathrm{CG}}^{\mathrm{bind}}\left(\theta \right)\right\rangle }_{\mathrm{CG}}]$$

Thus,

6 $$\nabla_{\theta }L_{reg-REM}=\beta {\left\langle \nabla_{\theta }U_{CG}\right\rangle }_{AA}-\beta {\left\langle \nabla_{\theta }U_{CG}\right\rangle }_{CG}-2\kappa \cdot \left(V_{0}-{\mathrm{V̅}}_{CG}^{bind}\left(\theta \right)\right)\cdot [{\left\langle \nabla_{\theta }V_{CG}^{bind}(\theta )\right\rangle }_{CG}-\beta {\left\langle V_{CG}^{bind}\left(\theta \right)\cdot \nabla_{\theta }U_{CG}\left(\theta \right)\right\rangle }_{CG}+\beta {\left\langle \nabla_{\theta }U_{CG}\left(\theta \right)\right\rangle }_{CG}{\left\langle V_{CG}^{bind}\left(\theta \right)\right\rangle }_{CG}]$$

Note that $\nabla_{\theta }U_{CG}\left(\theta \right)=\nabla_{\theta }V_{CG}^{bind}\left(\theta \right)$ if θ represents only intermolecular parameters. Implementation or application of this regularized version is as simple as the standard REM method, as it only requires the recording of the interaction energy between the binding partners and the evaluation of corresponding terms in eq 6.

### Atomistic Reference Simulations

Three model systems were considered, the HIV-1-protease dimer, the CA/SP1 immature HIV-1 Gag-lattice, and a complementary four base-pair DNA duplex (sequence: 5′-GCGC-3′). The 1hhp and 5l93 pdb-entries were used as starting structures for the protease and lattice system, respectively.^32,33^ The pdb2pqr server was used to identify protonation states that were assigned in the subsequent system-preparation with Gromacs.^34,35^ For these protein systems, the Charmm36m force field was employed.^36^ The starting structure for the DNA system was generated with the nab-module of the Amber18-package,^37^ and the DNA molecules were described with the Tumuc1 force field.^38^ All three systems were solvated with the TIP3P water-model and a salt concentration of 250 mM NaCl was implemented.^39^ The systems were energy-minimized in 5000 steps of steepest descents, and equilibrated for 500,000 steps with a time-step of 1 fs in the constant NVT-ensemble at the targeted reference temperatures (the same as in the following production runs) using a Nose-Hoover thermostat with a coupling constant of 1 ps.^40,41^ Final output-structures were used for production runs with an all-atom MD time-step of 2 fs. Reference temperatures were 310, 310, and 300 K for the protease-, CA/SP1-, and DNA-systems, respectively. The protease- and DNA-systems were simulated for at least 400 ns, and the CA/SP1-lattice for 200 ns. Coordinates were written out every 50,000 MD timesteps.

### Design of the CG Models

The protein systems were mapped to a resolution of 1 CG-bead per 4–5 amino acids. In total, each protease monomer was mapped to 25 beads, and each CA/SP1 monomer to 53 beads. The 4-bp DNA duplex was mapped to 32 beads, i.e., 4 beads per base. Mapping rules were derived with the Essential Dynamics Coarse-graining (EDCG) method^42^ in which atoms (indexed as *i* and *j*) are grouped into a CG-bead (indexed as I) to minimize the residual

7 $$\chi^{2}=\frac{1}{3N}\overset{N}{\underset{I=1}{\langle \sum }}\underset{i,j\in I}{\sum }{\left|\Delta r_{i}^{PC}-\Delta r_{j}^{PC}\right|}^{2}\rangle_{AA}$$

where $\Delta r_{i}^{PC}$ denotes the contribution Δ*r*_*i*_(*t*) = *r*_*i*_(*t*) - ⟨*r*_*i*_(*t*)⟩ in the subspace spanned by the principal components.

Analogously to ref 43, the effective potential energy for the CG-models was cast in the form

8 $$U\left(R^{N}\right)=U_{bond}\left(R^{N}\right)+U_{elec}\left(R^{N}\right)+U_{rep}\left(R^{N}\right)+U_{att}\left(R^{N}\right)$$

Here, *U*_*bond*_(*R* ^*N*^) represents intramonomer interactions that were described by heteroelastic network models (hENM) that consist of effective harmonic springs.^44^ Spring constants between CG beads (I, J) were iteratively updated (with learning-rate γ) to reproduce the statistics of the atomistic data:

9 $$\frac{1}{k_{IJ}^{n+1}}=\frac{1}{k_{IJ}^{n}}-\gamma_{hENM}\cdot \left({\left\langle R_{IJ}^{2}\right\rangle }_{CG}-{\left\langle R_{IJ}^{2}\right\rangle }_{AA}\right)$$

The EDCG and hENM procedures were carried out with the OpenMSCG software package.^45^

Nonbonded interactions of our CG models had the analytic forms:

10 $$U_{elec}\left(R^{N}\right)=\underset{I\in m_{\alpha },J\in m_{\beta >\alpha }}{\sum }\frac{Q_{I}Q_{J}}{4\pi \varepsilon_{0}\varepsilon_{r}R_{IJ}}\cdot \exp\left(-\kappa R_{IJ}\right)$$

11 $$U_{rep}\left(R^{N}\right)=\underset{I\in m_{\alpha },J\in m_{\beta >\alpha }}{\sum }A_{IJ}\cdot H\left(R_{IJ}^{rep}-R_{IJ}\right)\cdot \left[1+\cos\left(\frac{\pi R_{IJ}}{R_{IJ}^{rep}}\right)\right]+\frac{B}{{R_{IJ}}^{4}}$$

and

12 $$U_{att}\left(R^{N}\right)=\underset{I\in m_{\alpha },J\in m_{\beta >\alpha }}{\sum }\frac{C_{IJ}}{\sigma \sqrt{2\pi }}\exp\left(-\frac{{\left(R_{IJ}-R_{IJ}^{att}\right)}^{2}}{2\sigma^{2}}\right)$$

The dielectric constant ε_*r*_ was set to 17.5 and the inverse Debye-screening length κ to 1.274 nm^–1^.The CG-charges *Q*_*I*_,*Q*_*J*_ are the sums of atomistic charges that belong to each CG bead. The term *A*_*IJ*_ was chosen depending on the charges *Q*_*I*_ and *Q*_*J*_ in multiples of 10 kcal/mol (e.g., 10 kcal/mol if *sgn*(*Q*_*I*_) = *sgn*(*Q*_*J*_), and 100 kcal/mol if *Q*_*I*_·*Q*_*J*_ < −1). The term $R_{IJ}^{rep}$ was identified from the behavior of radial distribution functions. It was determined as the largest radius still smaller than the radius of the first peak of the radial distribution function as well as $p\left(R_{IJ}^{rep}\right)\leq 0.05p_{max}$ . In eq 11 *H* denotes the Heaviside-function, and the constant B was set to ∼6 kcal/mol·Å^4^. The $\frac{B}{{R_{IJ}}^{4}}$ term prevents $lim_{R_{IJ}⃗0}U\left(R^{N}\right)=-\infty$ for *sgn*(*Q*_*I*_) = −*sgn*(*Q*_*J*_), but otherwise does not affect the REM-training.

Slightly different choices were made for the multimeric CA/SP1 lattice system, where we set $R_{IJ}^{rep}=\frac{1}{2}\cdot \left(R_{I}^{gyr}+R_{J}^{gyr}\right)$ and *A*_*IJ*_ = 25.0 kcal/mol. This means a milder setting of repulsion that improved training stability of this multimeric system. For *U*_*att*_, σ was set to 0.2 nm and $R_{IJ}^{att}$ to the first peak in the radial distribution function, except for the CA/SP1 multimer, where $R_{IJ}^{att}$ was set to $min_{m_{\alpha ,}m_{\beta \neq \alpha }}{\left\langle R_{IJ,m_{\alpha }m_{\beta }}\right\rangle }_{AA}$ with *m*_α_ and *m*_β_ each denoting one of the 18 CA/SP1 monomers and I and J one of the 53 bead-types. The *C*_*IJ*_ parameters are learned during the REM-procedure. Note that all monomers have identical description and solvent-molecules were mapped-out in our CG-representation. The cutoff for all nonbonded interactions is 2.5 nm. All CG-simulations were performed with the OpenMM package.^46^ The number of CG-steps per REM iteration was 525,000, 1,525,000 and 1,250,000 for the protease, the CA/SP1 lattice and the DNA duplex, respectively. The time step of each simulation was 15 fs, and a Langevin-thermostat with reference temperatures of 310 K (protease, CA/SP1) and 300 K (DNA) was employed.

Mapping of atomistic trajectories and the REM-training were carried out with in-house written software.

## Results and Discussion

As a first example, we parametrized CG-models for the HIV-protease dimer comparatively, through training in a standard REM-approach and with our reg-REM method (Figure 1). We set $\kappa =\frac{0.1}{4.184}{\left(\frac{\mathrm{kJ}}{\mathrm{mol}}\right)}^{-2}$ and *V*_0_ = −25.0 kJ/mol for the training of this system with reg-REM. The choices of these parameters will be discussed later in the paper. During the atomistic MD simulation of this system (Figure 1A), the complex remained in the bound state. As discussed in the previous section and shown in Figure 1C, a standard REM approach is therefore agnostic of a lower bound for the binding affinity. Thus, the stability of the complex is continuously increased even when the training has already achieved an accurate description of the complex-structure as indicated by low RMSD-values (Figure 1E). From the practical point of view, one may argue that a single REM-iteration that reproduces the structure and the binding-affinity accurately is sufficient. However, even such behavior cannot be guaranteed with a standard REM approach and enforcing it would require substantial technical manipulation which does not provide a solution to the nature of the problem itself. Our reg-REM method instead accomplishes that. The training converges very well toward the desired binding affinity (average CG interaction energy between the binding partners, the dashed red line in Figure 1D). Importantly, this convergence does not interfere with the structural description of the complex (Figure 1F). Addition of the regularization term does not lead to partial unlearning of the complex-structure once the targeted binding-affinity is reached, but the sampled CG-structures still show a low RMSD-value with respect to the starting structure. Moreover, descriptions of the structure of the complex and distribution functions are also not degraded compared to the standard-REM procedure (compare Figures 1E,F and S1A). Our reg-REM approach yields multiple CG-models with an RMSD below 2 Å and binding affinities close to the targeted value of −25.0 kJ/mol, therefore demonstrating excellent training behavior. As shown in Figure S1B, reg-REM tends to soften attractive interactions after around 80 iterations, i.e., when a decent CG-model has already been learned. This indicates that initial training focuses on training the probability distribution, and later training on maintaining (regularizing) the binding affinity.

**Figure 1:**
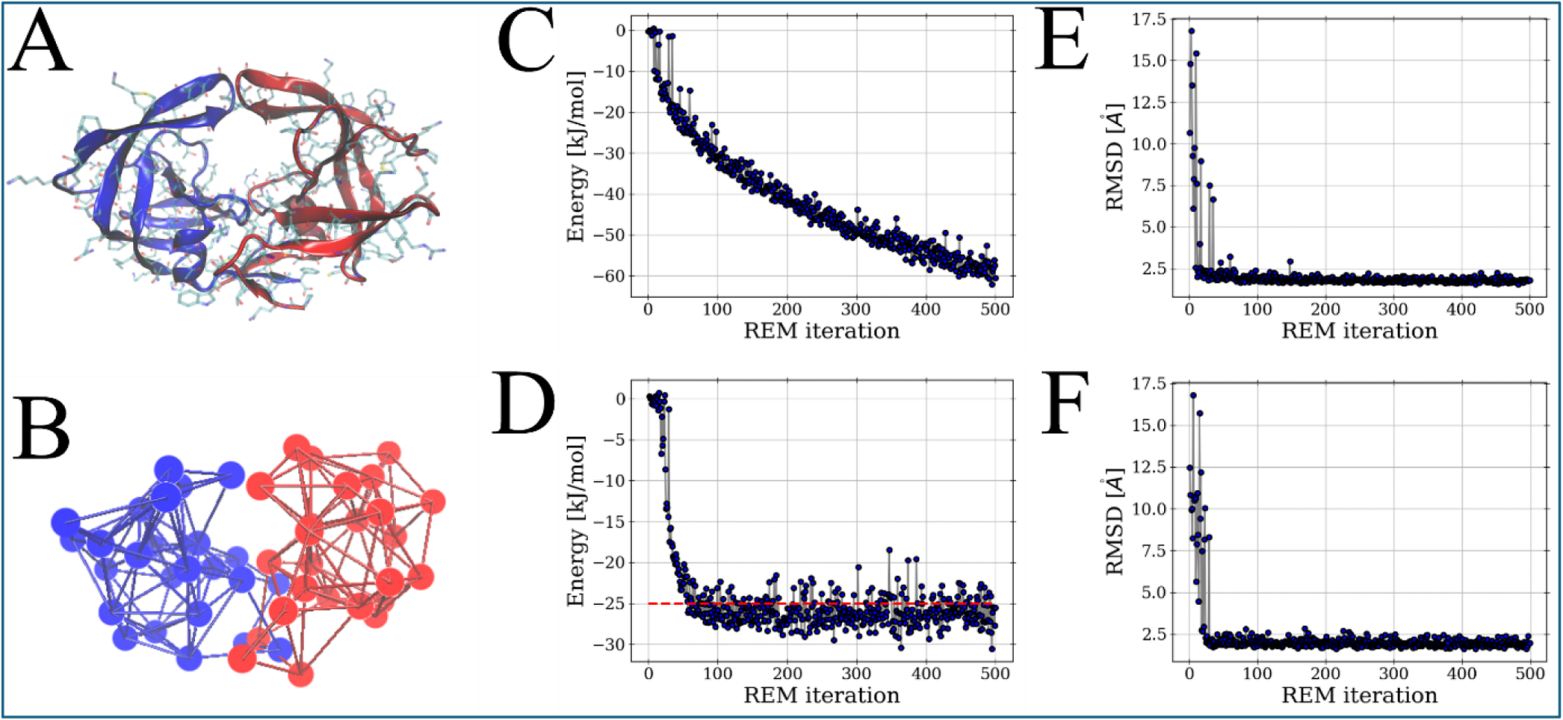
Training of a coarse-grained model for the HIV-protease dimer. The atomistic structure (A) of each binding partner is mapped to a coarse-grained representation consisting of 25 beads (B). The binding-energies are plotted as a function of iteration for the standard REM (C) and our reg-REM method (D). The dashed red line in D marks the empirical affinity *V*_0_. Average RMSD-values with respect to the native state per iteration are shown in (E,F) for the standard and regularized REM, respectively.

We have also trained the interactions for the CA/SP1 HIV-1 Gag complex consisting of 18 monomers similarly (Figure 2), but with *V*_0_, ${\mathrm{V̅}}_{CG}^{bind}$ , and the KL-terms acting on a specific pair within the complex (i.e., training is performed based on the interaction of two monomers). The iteratively updated parameters θ have then been used for all monomers. In this way, we have been able to train CG-models for the CA/SP1 monomers that capture the structure of the multimolecular complex very well (Figure 2C). Again, our reg-REM method also reproduces the targeted binding-affinity between two CA/SP1 monomers (Figure 2D) and does not show a noticeable trade-off between training structure and the input training affinity. We therefore argue that our proposed reg-REM method is also very suitable for the development of CG models for multimolecular complexes.

**Figure 2:**
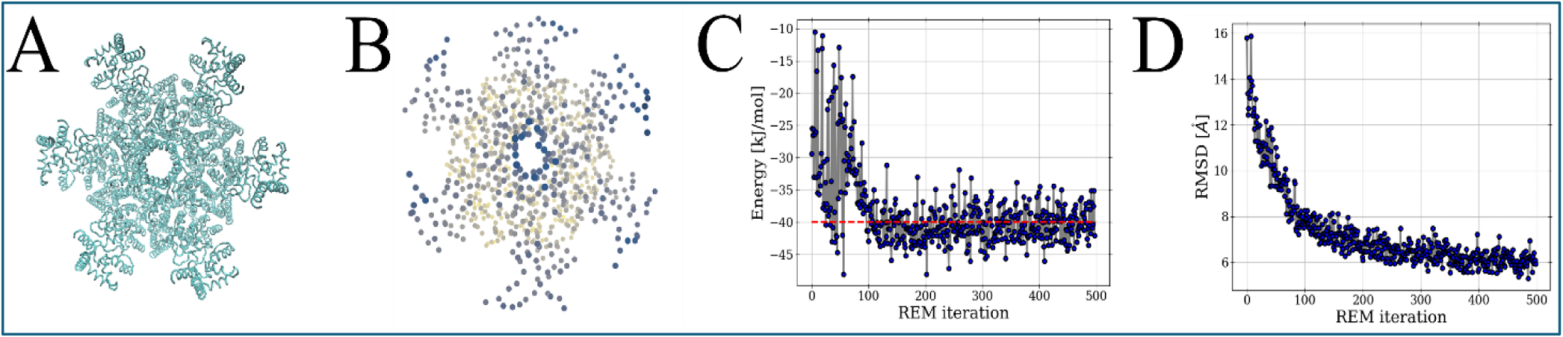
Training of a coarse-grained model for the immature CA/SP1 lattice. The 18 monomers form a hexameric structure (A: atomistic, B: coarse-grained). Learning the CG-potential parameters converges to the targeted binding affinity of −40 kJ/mol (C). Plotting the average RMSD with respect to the native structure reveals excellent learning of the complex structure (D).

Moreover, we show here that CG-models trained in this way are transferable to larger systems. We have applied our CG-model for the CA/SP1 monomers for an entire immature CA/SP1 virus-like particle (Figure 3).^33^ Throughout the simulation (30-million steps), the monomers stayed embedded in the virion lattice, which maintained its shape (Figures 3, S2, and S3). Thus, our reg-REM method can facilitate the training of robust and transferable CG-models.

**Figure 3:**
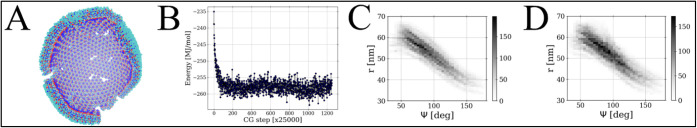
Reg-REM trained CA/SP1 model can be used for simulations of the entire virus-like particle consisting of 3180 monomers (A). The potential-energy as a function of simulation time reveals stability of the lattice and hence robustness of the parametrized CG-model (B). Relation between radial-distance (of monomers to mean-coordinate) and azimuthal orientation shows same characteristics at the beginning (C) and end of simulation (D), hence showing that the lattice-structure is maintained.

As another test-system, we have trained CG-models for a four base-pair DNA duplex, setting *V*_0_ = −16.0 kJ/mol (Figure 4). At such weak binding affinities, partial dissociation can already be expected during CG-simulations which can complicate the training of the model. This causes on average a slight overstabilization of ∼−3.0 kJ/mol for the trained models at $\kappa =\frac{0.1}{4.184}{\left(\mathrm{kJ}/\mathrm{mol}\right)}^{-2}$ (Figure 4C). Increasing the regularization strength to $\kappa =\frac{0.2}{4.184}{\left(\mathrm{kJ}/\mathrm{mol}\right)}^{-2}$ , trains a larger number of models closer to *V*_0_, but also increases the tendency of dissociation (Figure 4D). Both regularization-settings yield models that accurately describe the structure of the complexes (Figure 4E,F). Iterations with a RMSD of ∼8 Å exhibit fraying of the terminal base-pairs while the inner bases are still paired. Such behavior is not necessarily unreasonable, as local dissociation events are plausible during CG simulations at such low binding affinities and are in line with higher opening-rates of terminal base-pairs.^47,48^

**Figure 4:**
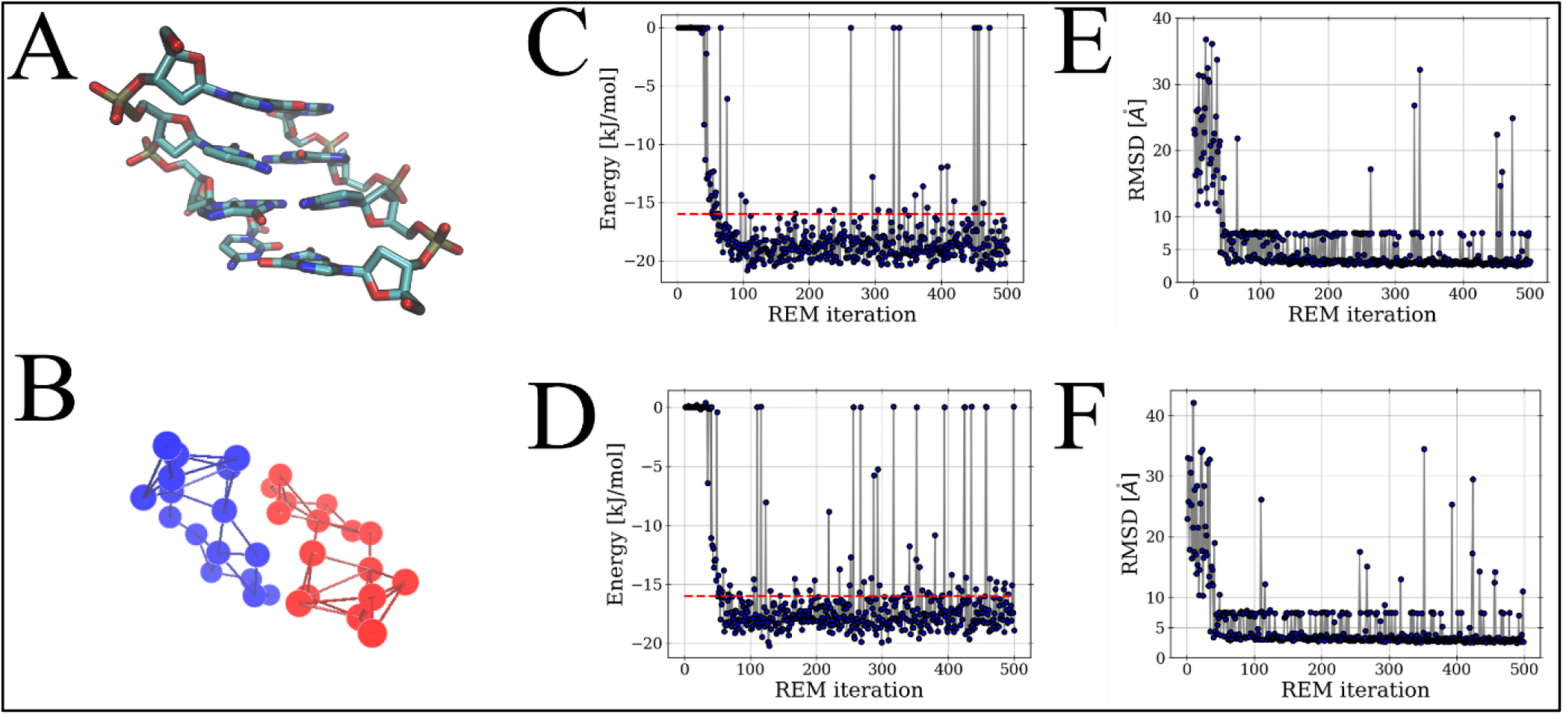
Reg-REM training of a 4 base-pair DNA duplex (A) mapped to 32 beads (B). The training was regularized by biasing the average binding energy to −16 kJ/mol using a regularization constant of $\frac{0.1}{4.184}{\left(\mathrm{kJ}/\mathrm{mol}\right)}^{-2}$ (C) and $\frac{0.2}{4.184}{\left(\mathrm{kJ}/\mathrm{mol}\right)}^{-2}$ (D). The corresponding mean-RMSD plots show multiple increases to ∼8 Å due to local fraying events (E,F).

We further tested the model by simulating one CG-model (iteration 494) at 320 K for 500 million CG-steps (Figure 5), since we expected dissociation events for a complex of such low binding-affinity at elevated temperature. In fact, the complex dissociated and correctly reformed multiple times during the simulation. This is an essential result in our study, as it manifests that our new approach allows one to coarse-grain molecular complexes so that the resulting models capture unbinding and rebinding into the correct complex structure. In the bound state, we again found transiently increased RMSD-levels due to fraying of terminal base-pairs. This behavior is reflected by higher distances between paired bases at the termini of the duplex compared to paired bases in the center, as terminal base-pair-distances fluctuate up to ∼15 Å and central base-pair-distances only up to ∼10 Å in the bound state (compare Figure 5B,C). Note that the CG potential energy constitutes a potential of mean force and hence a free energy. Thus, CG models are not simply temperature transferable.^14,49,50^ Nevertheless, the reg-REM approach might also be combined with a temperature-transferable framework, and the simulation at elevated temperature shows that our trained CG-models are not trapped in metastable states but un- and rebind frequently and capture the binding-structure very well. By capturing the empirical binding affinities, our reg-REM derived models effectively prevent overstabilization of molecular complexes and hence make it possible to capture some of the more intricate dynamics of large-scale molecular assembly which may require the dissociation of transient, intermediate states.^51,52^ For instance, one can coarse-grain DNA strands of various sequences with different binding affinities and simulate how they fold into higher-order structures with our reg-REM method.^53,54^

**Figure 5:**
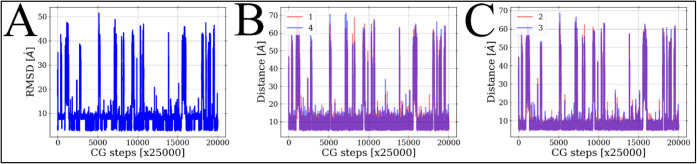
Simulation of a trained DNA CG-model at 320 K. As indicated by the RMSD-curve, the two DNA strands bind and unbind multiple times (A). Distance-plots between bases of the first as well as between bases of the fourth pair show increased fluctuations also in the bound state. These two base-pairs represent the terminal base-pairs of the DNA duplex (B). Inter base-pair distances of the two central base-pairs (labeled 2 and 3) reveal lower fluctuations and therefore higher stability compared to the terminal base-pairs (C).

We note that the required empirical input (*V*_0_, κ) for our reg-REM method may not always be obvious. In the following, we discuss the choice of these parameters. The binding energy *V*_0_ can be inferred from experimental or atomistic data. In our proof-of-concept reg-REM parametrizations, we trained different ranges of *V*_0_ to demonstrate that our reg-REM routine can be applied to cases of strong and weak binding. However, one needs to bear in mind that we bias toward the CG potential energy interaction in the reg-REM method. Although the CG potential energy effectively represents a free energy, “free volume” entropic terms of dissociation are missing but can be estimated^55^ and subtracted from the binding free energy to obtain an empirical estimate for *V*_0_.

As a rule of thumb, the reg-REM training becomes more challenging for weaker binding affinities, as these scenarios contrast with standard REM more strongly. For the protease-dimer, we used a weak binding affinity to show that reg-REM still performs well in this regime and therefore might also be used to parametrize weak-binding mutants.^56^ The binding affinity for the DNA duplex was chosen based on experimental, thermodynamic data for DNA duplex stability,^57^ and the training of the CA/SP1 lattice was carried out with a hypothetical estimate. Importantly, one can also perform atomistic free energy simulations to determine *V*_0_ more rigorously, for instance based on dissociation free energies from steered MD simulations (including restraints on orientational degrees of freedom). The approach presented in this paper, in contrast, is straightforward and does not require additional sampling.

For the choice of κ, we found that 0 ≤ ⟨∇_*θ*_ *U*_*CG*_(θ)⟩ < 2.0 *nm* ^–1^ due to our design of the trainable part of the potential energy (Figures S4–S6). Assuming small fluctuations in the binding energy implies that $\left|\nabla_{\theta }{\left\langle V_{CG}^{bind}\right\rangle }_{CG}\right|\gg \left|\beta {\left\langle V_{CG}^{bind}\cdot \nabla_{\theta }U_{CG}\right\rangle }_{CG}-\beta {\left\langle \nabla_{\theta }U_{CG}\right\rangle }_{CG}{\left\langle V_{CG}^{bind}\right\rangle }_{CG}\right|$ , and κ needs to be selected such that gradients due to the *D*_*KL*_ and regularization term are well balanced. It is suggested that a *D*_*KL*_-gradient of 0.5*βnm* ^–1^ shall be equal to a regularization gradient for $V_{0}-{\mathrm{V̅}}_{CG}^{bind}\left(\theta \right)=1\mathrm{kcal}/\mathrm{mol}$ , i.e.,

$0.5\beta nm^{-1}≅2\kappa \cdot \left(\mathrm{kcal}/\mathrm{mol}\right)\cdot \langle \nabla_{\theta }V_{CG}^{bind}{(\theta )\rangle }_{CG}$ . Approximating $\langle \nabla_{\theta }V_{CG}^{bind}{(\theta )\rangle }_{CG}\approx 1.0{\mathrm{nm}}^{-1}$ , we obtain $\kappa \approx \frac{0.1}{4.184}{\left(\frac{\mathrm{kJ}}{\mathrm{mol}}\right)}^{-2}$ . This choice led to good results for all of our test-systems.

## Conclusions

Bottom-up CG models have achieved substantial success and increasing recognition in the field of molecular simulation. The central dogma in bottom-up coarse-graining is to ensure thermodynamic consistency between the atomistic and coarse-grained ensembles; the development of a bottom-up CG model hence requires a preceding atomistic reference simulation. In this paper, we point out that atomistic reference simulations often can provide only limited sampling of the entire configuration space. This has substantial consequences for the coarse-graining of molecular complexes, as atomistic simulations generally reach too short time scales to observe multiple binding/unbinding events of large molecular complexes. In practice, conventional bottom-up coarse-graining (via FM or REM) is agnostic of a lower bound for the binding affinity of complexes and therefore leads to overfitting of the data sampled in the underlying atomistic simulation. Herein we solve this problem by adding a regularization term to the loss function of REM that acts on the average binding energy. By deriving update-rules for the parameters of the CG potential energy function, we demonstrate that CG models with both accurate binding-affinities and accurate structures can be parametrized with REM. We have applied this machine-learning-based coarse-graining concept to three different biomolecular systems. In all cases the successfully parametrized CG-models exhibit the targeted binding affinity and also describe the molecular complex structure accurately relative to the all-atom case. No noticeable trade-off between the training of the structure and the training of the binding affinity is observed. Thus, the new reg-REM method can be employed to coarse-grain models for protein complexes including oligomers, which can be used to simulate, e.g., entire protein-lattices such as HIV-1 virus-like particles. Analogously, we have also shown that smaller complexes with a weak binding affinity such as a four base-pair DNA duplex can be coarse-grained accurately with the reg-REM method. For example, simulation of such a CG-model at an elevated temperature displayed frequent binding and unbinding between the two DNA strands. Thus, our approach facilitates the development of CG models that exhibit a binding/unbinding dynamics.

In the future, the regularization approach can also be extended. For instance, the reg-REM method could be applied to neural network potentials,^58^ while devising a regularized Force-Matching routine is also of interest for the field of CG simulations. Finally, we emphasize that the reg-REM method is as easy to apply as the standard REM-procedure. As such, this approach can be used in the future to simulate assembly or disassembly of large biomolecular complexes as well as aid in the design of self-assembling nanomaterials.

## Supplementary Material



## Data Availability

All relevant code, input, and instruction files are available at 10.5281/zenodo.14579394.

## Notes

Probability distributions and learning behavior obtained from standard and regularized REM, snapshots of the virus-like particle, 2d distributions of orientational angles and radial distances of the CA/SP1 monomers, reference distributions of the expectation values for the partial derivatives of the potential energy with respect to the trainable parameters (PDF)
