# Supplementary material for: A Hybrid Bottom-Up and Data-Driven Machine Learning Approach for Accurate Coarse-Graining of Large Molecular Complexes
Source: J Chem Theory Comput. 2025 Apr 17;21(9):4846–54. doi: 10.1021/acs.jctc.5c00063 (PMC12268871; doi:10.1021/acs.jctc.5c00063)
Supplement: Supplementary file 1 [file ct5c00063_si_001.pdf]

Supporting Information for

A Hybrid Bottom-Up and Data-Driven Machine Learning Approach for Accurate Coarse-Graining of Large Molecular Complexes

Korbinian Liebl, and Gregory A. Voth*

Department of Chemistry, Chicago Center for Theoretical Chemistry, Institute for Biophysical Dynamics, and James Franck Institute, The University of Chicago, Chicago, IL 60637

Corresponding Author: gavoth@uchicago.edu

1. REM-trained Statistics and Parameters

As shown in Fig. S1A, standard REM and reg-REM show similar accuracy with respect to the distributions of intermolecular distances. The learning behavior of the parameters of the CG potential energy, however, shows substantial differences (Fig S1B). Several of the parameters increase monotonically when trained with standard REM which also results in a continuous increase in binding affinity between the partner molecules. In regularized REM, attractive interactions are often softened, once an accurate description of the molecular complex has been achieved.

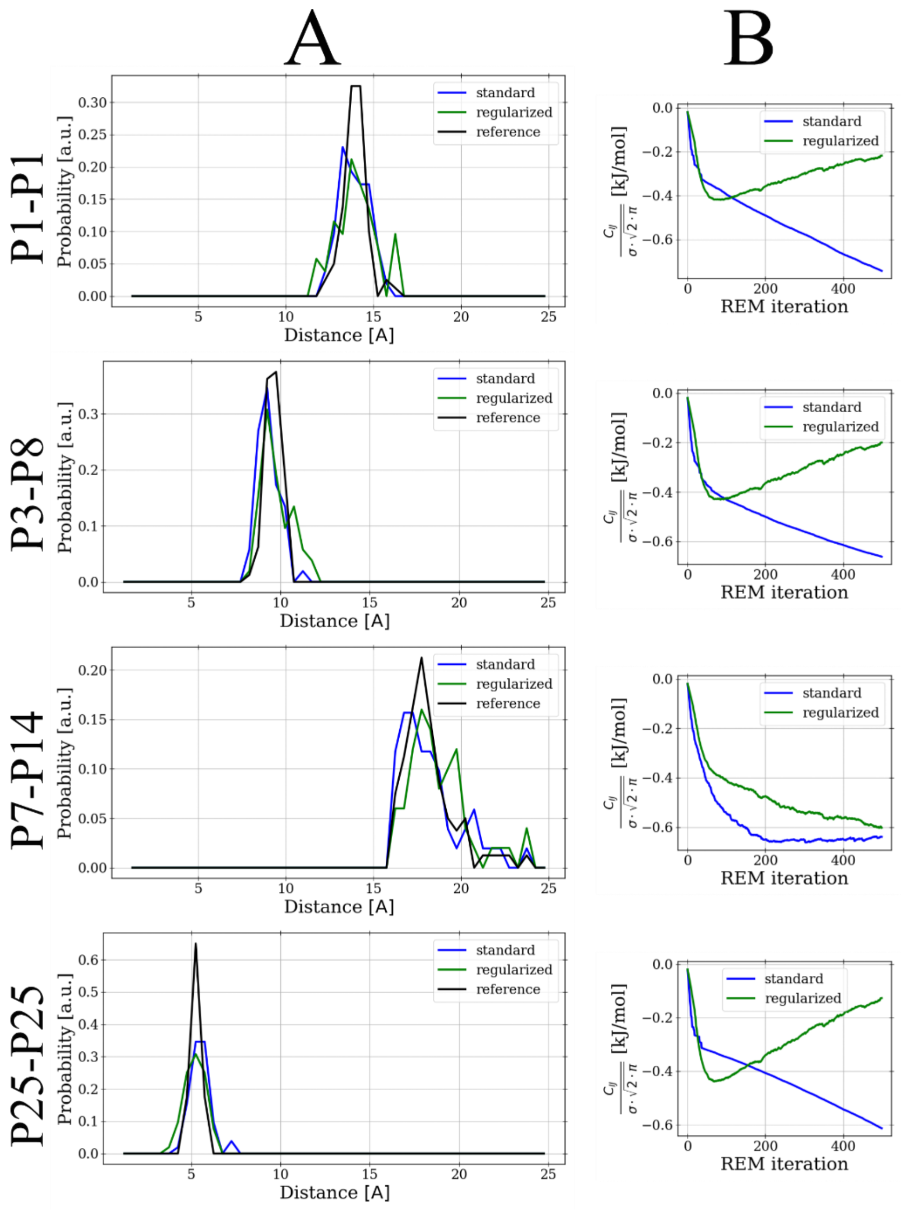

Figure S1: Trained probability distributions for inter-molecular distances between selected beads (A) show similar performance for reg-REM and standard REM. Learning behavior of the CG-parameters differs substantially between standard and regularized REM (B).

2. Virion Simulation

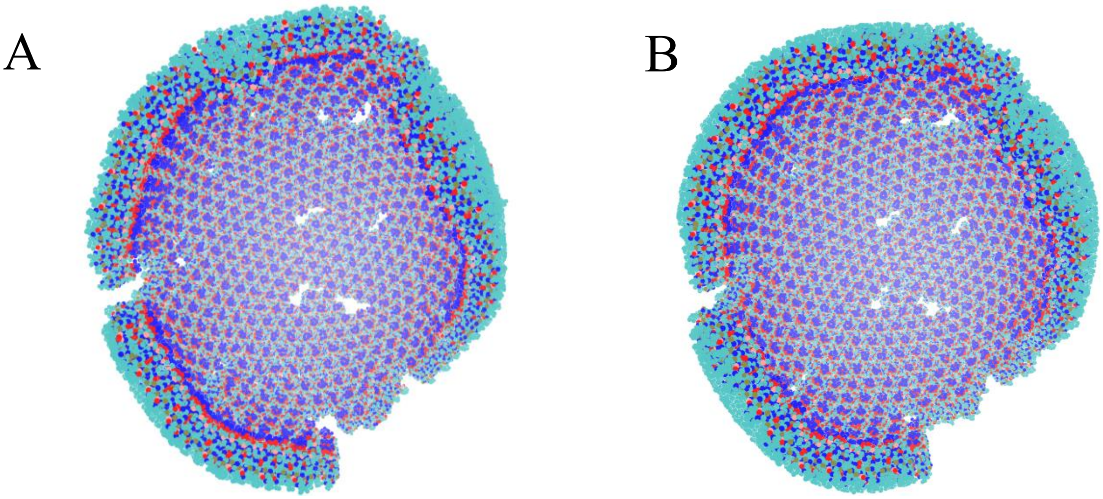

Figure S2: Starting structure for the immature CA/SP1 lattice (A) and final structure after simulation of 30mio CG-steps (B).

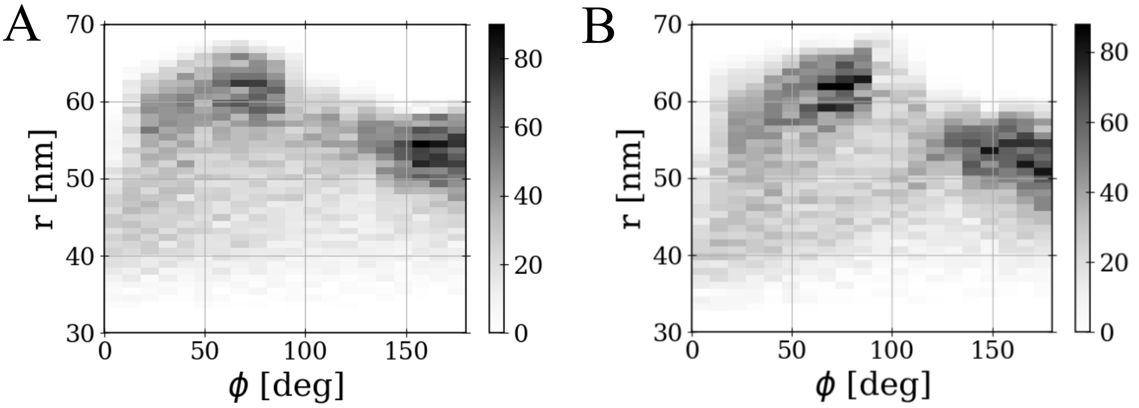

Figure S3: 2d-Distribution of orientational angles and radial distances of the CA/SP1 monomers for the starting structure (A) and the final structure (B). The reference points for the spherical coordinate systems are the cartesian coordinates averaged over all CA/SP1 monomers. The characteristic pattern is conserved, hence showing solid description of the virion-structure

3. Choice of reg-REM settings

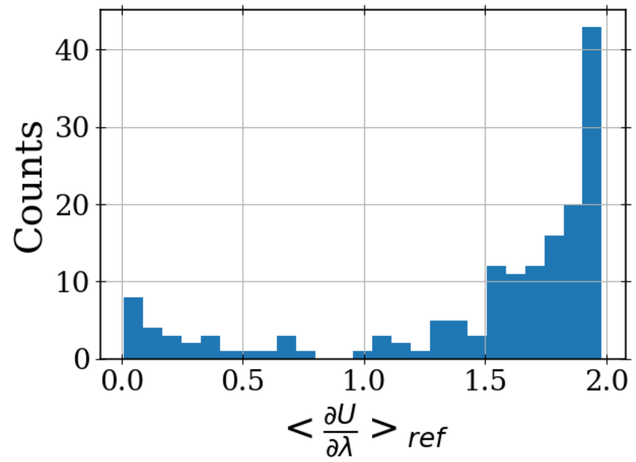

Figure S4: Distribution of expectation values for the partial derivatives obtained from the reference trajectory of the protease-dimer.

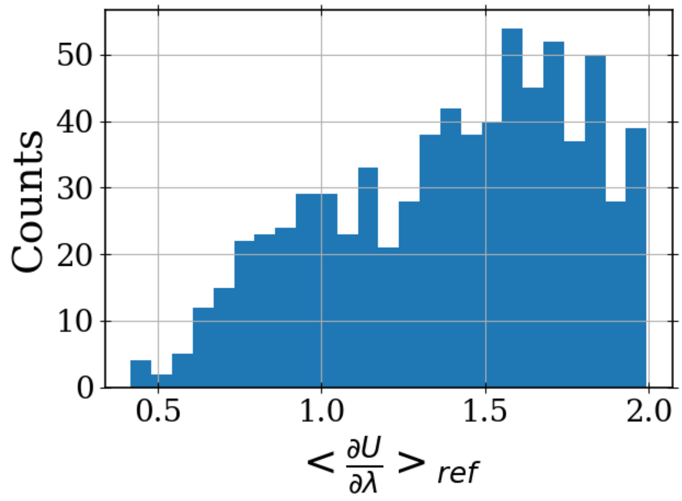

Figure S5: Distribution of expectation values for the partial derivatives obtained from the reference trajectory of the CA/SP1 lattice.

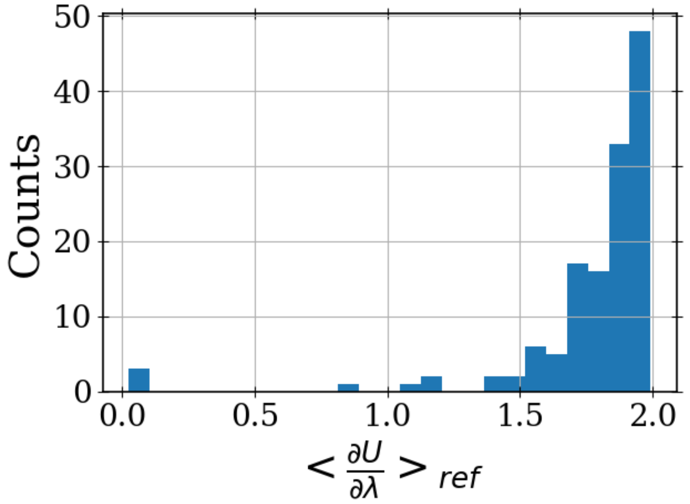

Figure S6: Distribution of expectation values for the partial derivatives obtained from the reference trajectory of the DNA-duplex simulation.
